# Supplementary material for: The interactome of intact mitochondria by cross-linking mass spectrometry provides evidence for coexisting respiratory supercomplexes
Source: Mol Cell Proteomics. 2017 Dec 8;17(2):216–32. doi: 10.1074/mcp.RA117.000470 (PMC5795388; doi:10.1074/mcp.RA117.000470)
Supplement: Supplemental Data [file supp_RA117.000470_133922_0_supp_23978_fzffwf.zip › spectra_annotation/mito_DR_spectra_annotation/456-1-5-1-4-1.pdf]

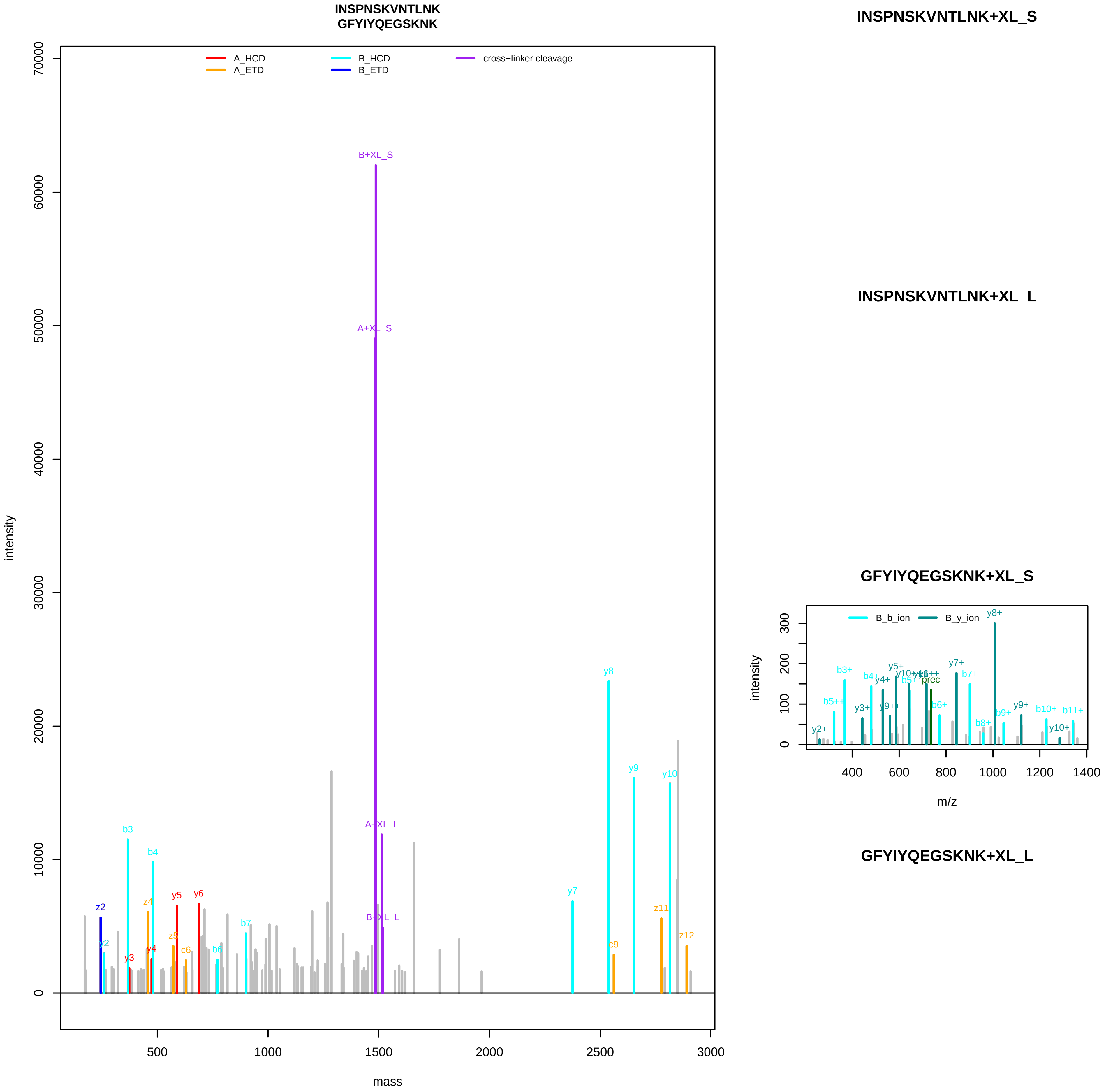
INSPNSKVNTLNK
GFYIYQEGSKNK
A_HCD
A_ETD
B_HCD
B_ETD
cross−linker cleavage
intensity
mass
B+XL_S
A+XL_S
A+XL_L
B+XL_L
z2
y2
b3
y3
b4
y4
z4
z5
y5
c6
y6
b6
b7
y7
y8
c9
y9
y10
z11
z12
INSPNSKVNTLNK+XL_S
INSPNSKVNTLNK+XL_L
GFYIYQEGSKNK+XL_S
B_b_ion
B_y_ion
m/z
y2+
b5++
b3+
y3+
b4+
y4+
y9++
y5+
y10++
b5+
prec
y7+
b7+
b6+
b8+
y8+
b9+
y9+
b10+
y10+
b11+
GFYIYQEGSKNK+XL_L
